# Vascular-directed responses of microglia produced by methamphetamine exposure: indirect evidence that microglia are involved in vascular repair?

**DOI:** 10.1186/s12974-016-0526-6

**Published:** 2016-03-12

**Authors:** John F. Bowyer, Sumit Sarkar, Karen M. Tranter, Joseph P. Hanig, Diane B. Miller, James P. O’Callaghan

**Affiliations:** Division of Neurotoxicology, National Center for Toxicology/FDA, Jefferson, AR 72079 USA; Center for Drug Evaluation and Research/FDA, Silver Spring, MD 20993 USA; Health Effects Laboratory Division, Centers for Disease Control and Prevention, National Institute for Occupational Safety and Health, Morgantown, WV 26505 USA; National Center for Toxicological Research/FDA, 3900 NCTR Road, HFT-132, Jefferson, AR 72079 USA

**Keywords:** Microglia, Vascular damage, Methamphetamine, Amphetamine, Neurotoxicity, Hyperthermia, Hypertension

## Abstract

**Background:**

Brain microglial activations and damage responses are most commonly associated with neurodegeneration or systemic innate immune system activation. Here, we used histological methods to focus on microglial responses that are directed towards brain vasculature, previously undescribed, after a neurotoxic exposure to methamphetamine.

**Methods:**

Male rats were given doses of methamphetamine that produce pronounced hyperthermia, hypertension, and toxicity. Identification of microglia and microglia-like cells (pericytes and possibly perivascular cells) was done using immunoreactivity to allograft inflammatory factor 1 (Aif1 a.k.a Iba1) and alpha M integrin (Itgam a.k.a. Cd11b) while vasculature endothelium was identified using rat endothelial cell antigen 1 (RECA-1). Regions of neuronal, axonal, and nerve terminal degeneration were determined using Fluoro-Jade C.

**Results:**

Dual labeling of vasculature (RECA-1) and microglia (Iba1) showed a strong association of hypertrophied cells surrounding and juxtaposed to vasculature in the septum, medial dorsal hippocampus, piriform cortex, and thalamus. The Iba1 labeling was more pronounced in the cell body while Cd11b more so in the processes of activated microglia. These regions have been previously identified to have vascular leakage after neurotoxic methamphetamine exposure. Dual labeling with Fluoro-Jade C and Iba1 indicated that there was minimal or no evidence of neuronal damage in the septum and hippocampus where many hypertrophied Iba1-labeled cells were found to be associated with vasculature. Although microglial activation around the prominent neurodegeneration was found in the thalamus, there were also many examples of activated microglia associated with vasculature.

**Conclusions:**

The data implicate microglia, and possibly related cell types, in playing a major role in responding to methamphetamine-induced vascular damage, and possibly repair, in the absence of neurodegeneration. Identifying brain regions with hypertrophied/activated microglial-like cells associated with vasculature has the potential for identifying regions of more subtle examples of vascular damage and BBB compromise.

**Electronic supplementary material:**

The online version of this article (doi:10.1186/s12974-016-0526-6) contains supplementary material, which is available to authorized users.

## Background

Microglia activation responses in connection with neurotoxic insults have been investigated for now over 30 years [1–8]. Microglial responses to METH and amphetamine (AMPH) damage to dopaminergic axons and nerve terminals in the striatum [9, 10] and neurodegeneration in the parietal cortex, thalamus, and hippocampus and other limbic regions [11–13] were first identified over 20 years ago. In most cases, it has now been shown that microglia are not involved in producing or exacerbating the neurodegeneration, but responding to it [2, 3, 11–13]. This has been observed for dopaminergic terminal damage in the striatum due to amphetamines or MPTP [14–16]. Most clearly, it was shown that you can block the microglial response without affecting the degree of neurodegeneration, indicating parallel processes [14]. It has been known for some time that microglia play a role in scavenging degenerating neurons [17]. Although the origin and development of microglia in the brain has been elucidated [18], the role of microglia in disease and the neurotoxic process is continually being reevaluated and debated [8, 14, 19–23].

Significant microglia activation can occur just due to systemic signals circulating in the blood such as bacterial inflammagens, lipopolysaccharide (LPS), or damage-associated molecular proteins [24] (DAMPs) that do not cause neurodegeneration [14, 23]. This raises the possibility that, in regions of the brain where subtle or limited blood-brain barrier (BBB) breakdown or vascular leakage occurs, the activation of microglia and related cells that are adjacent to brain vasculature will occur. Histological signs of BBB breakdown in the septum and medial hippocampus of mouse and rat occur after a single very high dose of METH that can be the “driving force behind” the significant and prolonged signs of seizure activity [25, 26]. As well, microglial activation via isolectin B4 identification surrounding the vasculature is prominent, particularly in the hippocampus of the mouse brain. However, there is also a significant amount of neurodegeneration in these regions raising the possibility that the microglial response observed may be solely due to neurodegeneration.

Studies have indicated that other paradigms [21, 22] used to produce METH or AMPH neurotoxicity in rat or mouse can produce multiple focal areas (0.25 to 1 mm^2^) of vascular leakage within the thalamus and limbic regions that appear when body temperatures are ≥41.7 °C [27, 28]. However, behavioral signs of seizure activity are much less pronounced or absent in the paradigm in which four injections (ranging from 5 to 10 mg/kg) are spaced over a 6-h period [26]. As well, although neurodegeneration occurs in the thalamus, in this paradigm, there is usually only minor axonal damage in the more medial and ventral septum and almost no evidence of neurotoxicity in the hippocampus. Therefore, in the present study, we chose this type of dosing paradigm [29] now commonly used to produce METH or amphetamine toxicity, which consisted of giving four injections of 5, 7.5, 10, and 10 mg/kg of METH with 2 h in between each injection to see if there was significant microglial activation in the septum, hippocampus, and thalamus and associated vasculature.

## Methods

### Animals

This study was carried out in accordance with the declaration of Helsinki and the Guide for the Care and Use of Laboratory Animals as adopted and promulgated by the National Institutes of Health. The use of animal testing in this study was done under protocols E7295 and E7519 (issued to John Bowyer) that were approved by the NCTR institutional animal care and use committee (IACUC) which is fully accredited (Food and Drug Administration—National Center for Toxicological Research Accreditation #A4310-01) by NIH-OLAW. Sixty-five-day-old (300–350 g) male Sprague-Dawley rats, ninety-five total, were obtained from the Charles River Laboratories [Crl:CD(SD)]. Upon arrival at NCTR, they received tail tattoos for identification. Prior to testing, rats were housed 2 per cage with food and water available ad libitum. Rats were housed on a daily 12-h light cycle with lights on at 6:00 am and off at 6:00 pm. During housing, the temperature (23 °C) and humidity (53 %) were controlled. The rats were tested between 85 and 90 days (12 weeks) of age.

### Administration of METH

Dosing with METH commenced between 7:30 and 8:00 a.m. and ended between 1:30 and 2:00 p.m. Rats were individually housed during testing, and their wood chip bedding was replaced with absorbent pads to keep the METH animals from ingesting the wood chips. Animals were given either four injections (each spaced by 2-h intervals) of 1 ml/kg saline s.c. or four injections of METH (5, 7.5, 10, and 10 mg/kg s.c. spaced at 2-h intervals) at an environmental temperature of 22.5 °C. The d-METH (d-methamphetamine HCl; Sigma-Aldrich, St. Louis, MO) was dissolved in normal saline. The behavior and body temperature of all animals in all groups were monitored at least every hour during testing until at least 3 h after the last/fourth injection (time of sacrifice). A flexible probe was inserted approximately 7 cm into the colon to measure body temperature. The animals given saline at 22.5 °C remained normothermic. The lethal effects of hyperthermia in the METH group, when body temperatures exceeded 41.6 °C, were prevented by placing the animals unrestrained on crushed ice for 15 to 30 min in a clean, wood chip-free cage to allow their temperatures to drop below 40.0 °C.

### Animal perfusions and preparation for histological analysis

The perfusion process necessary for histological processing was initiated by giving the rats a lethal dose of approximately 150 to 300 mg/kg i.p of pentobarbital and 20 to 40 mg/kg sodium phenytoin derived from a mixture of Euthasol® from Vibrac AH, Inc., containing in 390 mg/ml pentobarbital and 50 mg/ml sodium phenytoin diluted 1:4 in sterile normal saline. When the rat’s respiration had waned to that barely detectable, it was perfused with 50 ml saline followed by 200 ml of 4 % formaldehyde in 0.1 M sodium phosphate buffer (pH 7.4). Details of the perfusion process have been previously reported [11]. Brains were postfixed for 36 h in 4 % formaldehyde in 0.1 M sodium phosphate buffer and then transferred to a 20 % sucrose and 0.1 M sodium phosphate buffer for at least 3 days. Sectioning of the brain to produce 25 to 30 μm sections was performed either using unfrozen fixed brain as previously described [11] or 20- to 25-μm-thick sections using a cryostat. For cryostat sectioning, the brain was removed from the sucrose and rapidly frozen in crushed/powdered dry ice. It was then embedded in OCT compound (Electron Microscope Science, Hatfield, PA, USA) and mounted on a Leica Cryostat for cutting coronal sections 20- to 25-μm thick from +1.2 to −6.0 relative to the Bregma [30]. Approximately half of the sections were stored at 4 °C in 0.1 M phosphate buffer pH 7.4 containing 4 % formalin until histological processing. The remaining sections were in stored 0.1 M phosphate buffer pH 7.4 containing 0.08 % sodium azide at 4 °C to avoid loss of immunoreactivity or antigens present in the tissue.

### Immunohistological and Fluoro-Jade C labeling

#### Fluoro-Jade C labeling

Methods of Schmued et al. [31] with minor modifications were used to detect degenerating neurons, dendrites, axons, and terminals in the forebrain using Fluoro-Jade C (FJc). The modifications made results in a slightly higher background, which increases the possibility of detecting a false positive signal, but this was deemed necessary to enable dual labeling of FJc with DAB immune-labeling of microglia or vasculature. Sections, either unprocessed or previously immune-labeled, were mounted on gelatin (Sigma, 300Bloom)-coated slides and dried at 50 °C. They were then transferred sequentially through solutions of 100 % ethanol (8 min), 95 % ethanol (2 min), 70 % ethanol (2 min) and two times double-deionized water (2 min). They were then immersed in double distilled water containing 0.06 % potassium permanganate (4 to 6 min for immune-labeled section or 8 to 10 min for unprocessed sections, followed by two times double-deionized water (2 min). Labeling was performed in a 0.0001 % FJc (Histo-Chem, Jefferson, AR, USA) in 0.1 % acetic acid and distilled water solution followed by rinsing in double distilled water three times (1 min per wash) to remove excess label. Slides were then rapidly air-dried, xylene-cleared and cover-slipped with DPX (Fluka/Sigma, St. Louis, Mo, USA) mounting media. In instances where sections were double labeled with FJc and Iba-1 or Cd11b, the Iba-1 and Cd11b labeling with DAB was performed first after which the sections were mounted on the gelatin slides and processed with FJc. The only modification of the procedure for double labeling was that the sections were exposed to the permanganate solution for only 4 min.

#### Immunohistochemical labeling

For immune-reactive labeling of brain sections, either single or double, the following procedures were used. In all steps the sections, whether mounted on slides or free-floating in incubation wells, were gently agitated on an orbital shaker. Sections were first washed 15 min in 0.1 M phosphate buffer pH 7.4. In cases where diaminobenzedine (DAB) was used as the chromophore, the sections were then incubated in phosphate buffer containing 0.3 % H_2_O_2_ for 30 min to destroy the endogenous peroxidases. From this point on, except with the last step of DAB processing the incubation and washing solutions used consisted of 0.1 M phosphate buffer pH7.4 containing 0.4 % Triton X-100 in double distilled water. The sections were subsequently washed for 5 min. After a 30-min preincubation in 4 % normal serum (from the animal that the secondary antibody was raised in), the sections were incubated in normal serum and the primary antibody for 1 to 2 h followed 18 to 24 h at 5 °C. The sections were then washed three times for 10 min and incubated in secondary antibody for 1 to 2 h. In cases where only single labeling for Iba1 was performed, the signal was amplified using the avidin and biotinylated horseradish peroxidase macromolecular complex (ABC, Vector Laboratories, Burlingame, CA) and visualized with 0.4 mg/ml of 3,3′-diaminobenzidine (DAB) in 50 mM Tris-buffer. The following primary antibodies used were rabbit anti-Iba1, Wako Inc., Japan; rabbit anti-RECA-1 from Abcam, Cambridge, USA; and mouse anti-rat Cd11b, Abd Serotek, USA. The secondary antibodies used were biotinylated goat anti-rabbit, Vector Laboratories, Burlingame, CA, and biotinylated donkey anti-rabbit, Jackson Immunoresearch, Philadelphia, PA).

#### Combined RECA-1 DAB and Iba1 TRITC fluorescent immunohistochemistry

DAB immunolabeling of RECA-1 was performed first, as described above, using a biotinylated goat anti-rabbit secondary antibody. Following DAB reaction, the sections were washed 3× (15 min/ wash) in buffer. Sections were then pre-incubated with 10 % normal horse serum (GIBCO, USA) for 30 min and then incubated with the primary rabbit anti-Iba1 antibody for 2 h at room temperature followed by 18 h at 5 °C. Sections were then washed 3× followed by incubation in a secondary biotinylated donkey anti-rabbit IgG (1:200; Jackson Immunoresearch, Philadelphia, PA) for 1 to 2 h. Subsequently, they were washed three times and incubated in a Streptavidin-TRITC (1:250, Jackson Immunoresearch, Philadelphia, PA) for 2 h at room temperature. Sections were then washed two times (5 min per wash) and mounted on slides dried. Finally, the slides were cleared in xylene and cover-slipped with DPX mounting medium. Methods very similar to above were used for DAB immunolabeling of Iba1 and TRITC immunolabeling of GFAP to visualize microglia and astrocyte morphology after METH in Fig. 9. GFAP immunolabeling was performed using a polyclonal rabbit anti-GFAP primary antibody (Dako/Agilent Technologies, USA).

#### Combined RECA-1 and Cd11b immunohistochemistry

Co-localization of integrin alpha M (complement component 3 receptor 3 subunit) a.k.a Cd11b was also used to identify macrophages in association with vascular endothelia. DAB immunolabeling of CD11b was performed first described in the previous section using a biotinylated donkey anti-mouse secondary antibody. Following DAB reaction, the sections were washed for 5 min in Tris-buffer followed by washing in Tris-buffer saline (TBS). Sections were then pre-incubated with 10 % normal horse serum (GIBCO, USA) made in TBS for 30 min and then incubated with the primary mouse RECA-1 antibody for 24–48 h at 4 °C. After 48 h, tissue sections were rinsed in TBS containing Triton-X for 3–5 min for three times followed by incubation in biotinylated secondary antibody (1:200 dilution) for 2–3 h at RT using TBS as antibody diluent. Then sections were washed in TBS containing Triton-X for 3–5 min, and incubated in Streptavidin-Alkaline Phosphatase (1:200; Promega, USA) for 2 h are RT. Then tissue sections were rinsed in TBS containing Triton-X for 3–5 min for three times. Sections were incubated in substrate for alkaline phosphatase (nitro blue tetrazolium; ready to use) for 1–2 min, and color reaction was monitored under microscope. Once the reaction was complete, the tissue sections were rinsed in TBS containing Triton-X for 3–5 min for three times. Subsequently, sections were mounted in distilled water containing 0.1 M PB, dried in warmer, cleared in Xylene, cover-slipped using special mounting medium (H-5000; Vector laboratories), and dried overnight under the hood.

#### Combined Cd11b and IBA-1 immunohistochemistry

Co-localization of integrin alpha M (complement component 3 receptor 3 subunit) a.k.a Cd11b was also used to identify macrophages in association with another microglial marker IBA1. DAB immunolabeling of CD11b was performed first described in the previous section using a biotinylated donkey anti-mouse secondary antibody. Following DAB reaction, the sections were washed for 5 min in Tris-buffer followed by washing in Tris-buffer saline (TBS). Sections were then pre-incubated with 10 % normal horse serum (GIBCO, USA) made in TBS for 30 min and then incubated with the primary rabbit IBA-1 antibody for 24–48 h at 4 °C. After 48 h, tissue sections were rinsed in TBS containing Triton-X for 3–5 min for three times followed by incubation in biotinylated secondary antibody (1:200 dilution) for 2–3 h at RT using TBS as antibody diluent. Then sections were washed in TBS containing Triton-X for 3–5 min, and incubated in Streptavidin-Alkaline Phosphatase (1:200; Promega) for 2 h are RT. Then tissue sections were rinsed in TBS containing Triton-X for 3–5 min for three times. Sections were incubated in substrate for alkaline phosphatase (nitro blue tetrazolium; ready to use) for 1–2 min, and color reaction was monitored under microscope. Once the reaction was complete, the tissue sections were rinsed in TBS containing Triton-X for 3–5 min for three times. Subsequently, sections were mounted in distilled water containing 0.1 M PB, dried in warmer, cleared in Xylene, cover-slipped using special mounting medium (H-5000; Vector laboratories), and dried overnight under the hood.

#### Visualization

Histological examination of the slide-mounted tissue was examined using a Nikon epifluorescent microscope 80i (Nikon Instruments Inc., Melville, NY) and an X-Cite 120 LED light source (Excelitas Technologies®, Waltham, MA). The following sets of filters were used for visualization fluorescent labeling in conjunction with the histochemical or immunohistological labeling: TRITC excitation of 533–553 nm and an emission of 573–613 nm and FITC excitation of 464.5–499.5 and an emission of 516–556 nm. Combinations of these filters resulted in negligible cross-talk between the signals from individual fluorochromes, such that there was minimal to no observable bleed-through between any of the three signals. Visualization of DAB-labeled sections was done with incandescent illumination. All photomicrographs of the images were taken using a Nikon DS-Ri1 using the NIS software from Nikon.

## Results

Seventeen of the 19 animals dosed with METH and sacrificed at the 3 days time point had body temperatures ≥40.0 °C for 4 h or more and at least two episodes of body temperatures ≥41.7 °C requiring hypothermic intervention/cooling (Table 1). The remaining two animals had body temperatures ≥40.0 °C for 4 h or more but their peak body temperatures did not reach 41.7 °C, and thus they did not requiring cooling. The survival rate was approximately 90 % for the METH-treated animals. As expected with this type of dosing paradigm originally developed to in rodent to produce pronounced and prolonged striatal dopamine depletions and terminal damage/destructions due to METH (or amphetamine) exposure [29, 32–35], histological evidence of dopamine terminal damage and destruction were pronounced (data not shown).

**Table 1:** Summary of hyperthermia, reactive microglia, and neurodegeneration after METH exposure

				Brain regions							
				Septum		Hippocampus		Thalamus		Parietal cortex	
Rat ID	Time point (days)	Peak BT (°C)	Times BT ≥ 41.7 °C	^a^FJc labeling	^b^Activated microglia	^a^FJc^+^ labeling	^b^Activated microglia	^c^FJc labeling	^b^Activated microglia	^c^FJc labeling	^b^Activated microglia
FG278	3ys	41.6	−	−	−	−	−	−	−	1	−
FG279	3	41.0	−	−	−	−	−	−	−	1	−
FG230	3	42.0	2×	−	−	−	−	−	−	4	−
FG288	3	41.9	3×	−	−	−	−	20	++	2	−
FG227	3	42.2	3×	+	+++	−	+++	17	+	8	+
FG268	3	42.6	3×	−	−	−	−	3	−	10	+
FG298	3	42.0	3×	−	+	−	+	5	+	10	+
FG289	3	42.7	4×	−	−	−	+	38	+++	9	+
FG269	3	42.6	4×	−	+	−	+	27	++	2	−
FG228	3	42.3	5×	+	+++	−	++++	132	++++	7	+
FG267	3	42.3	5×	+	++++	−	++++	37	+++	4	+
FG291	3	42.2	5×	+	++++	−	++++	91	++++	12	+
FG311	3	42.4	5×	+	+++	−	++	53	+++	7	+
FG258	1	41.9	2×	−	−	−	−	0	−	3	−
FG259	1	42.3	3×	−	−	−	−	6	−	6	−
FG260	1	42.1	3×	−	−	−	−	0	−	3	−
FG310	1	42.2	4×	+?	+?	+?	+?	53	−	6	−

Data relating to neurodegeneration or microglia activation for the four control animals is not shown since it was all negative. The correlation between the numbers of times body temperature was ≥41.7 °C, and the relative number of activated microglia within a region was determined using the Spearman rank-order correlation method. The correlation coefficients were as follows: septum, *r* = 0.781, *p* = 0.0008; hippocampus, *r* = 0.850, *p* < 0.00001; thalamus, *r* = 0.927, *p* < 0.00001; parietal cortex, *r* = 0.652, *p* < 0.014. The correlation between the numbers of times body temperature was ≥41.7 °C and the relative number of FJc within a region was determined using the Pearson product-moment correlation. The correlation coefficients were as follows: thalamus, *r* = 0.714, *p* = 0.0061; parietal cortex, *r* = 0.546, *p* = 0.054 not significant

^a^FJc positive fibers (>10 per 25-μm section per hemisphere) observed in the medial septum

^b^Relative number of activated Iba1 immunoreactive cells/microglia per 25-μm section per hemisphere such that per region per hemisphere: (−) indicates <5 cells per region; (+) 5 to 20 cells; (++) 20 to 100 cells; (+++) >100 cells

^c^FJc positive cells per 25-μm section per hemisphere observed in thalamus or parietal cortex

Iba1 immunoreactivity, which is an often used biomarker for microglia [36], was used to detect microglia in the septum, medial hippocampus, thalamus, and parietal cortex. The presence of activated microglia and FJc-positive (FJc^+^) cells in four brain regions are shown in Table 1. Both activated and resting microglia can be clearly and distinctly labeled by Iba1 immunoreactivity; those that are activated have a greatly enlarged soma, and often, the processes emanating from the soma are enlarged but of lesser length. For animals sacrificed 3 days after METH exposure, the number of Iba1 immunoreactive activated (based on criteria stated above) microglia in the dorsal lateral septum, anterior medial hippocampus, and the intralaminar, ventrolateral (VL), and ventromedial (VM) thalamus nuclei correlate positively with the number of times the body temperature of an animal exceeded 41.7 °C (see Table 1 for details). As well, the number of FJc^+^ cells in the thalamus and FJc^+^ fibers in the septum correlated with episodes of body temperatures ≥41.7 °C, which was not the case for the number of FJc^+^ cells present in the parietal cortex.

**Fig. 1:**
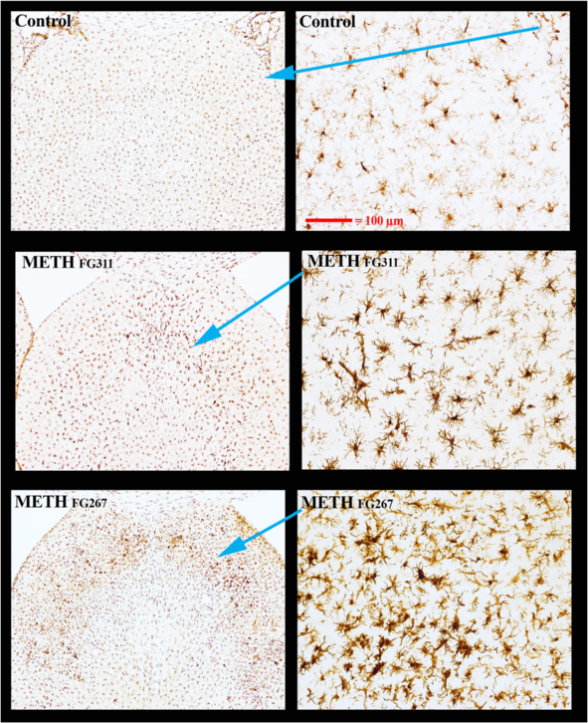
Microglia activation in the septum at 3 days after METH. Septal microglia were DAB immunolabeled for IBA1 and are shown at low (*left-most panels*) and high magnification (*right-most panels*). Magnification for the three right-hand panels was the same (see *magnification bar* on the top for reference). The *blue arrows* show where the regions of high magnification reside in the low magnification panels

**Fig. 2:**
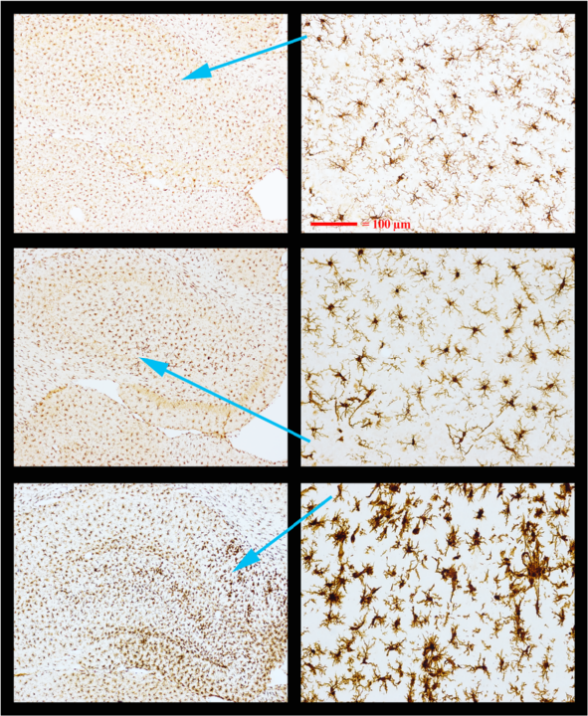
Microglia activation in the hippocampus at 3 days after METH. The microglia located in the more rostral and dorsal-medial regions of the hippocampus were DAB immunolabeled for IBA1 and are shown at low (*left-most panels*) and high magnification (*right-most panels*). Magnification for the three right-hand panels was the same (see *magnification bar* on the top for reference). The *blue arrows* show where the region of high magnification resides in the low magnification panels

The morphology of the majority of the activated microglia in the parietal cortex was of the hypertrophied spherical soma (with concomitant loss of long fibrous looking processes) type previously identified as being associated with degenerating neurons [2] (data not shown). However, there were some activated microglia in this region with the morphology of the predominant type seen in the septum and hippocampus (see below). The total number of activated microglia in the more dorsal and lateral septum (Fig. 1 and Additional file 1: Figure S1A) and anterior medial hippocampus (Fig. 2 and Additional file 1: Figure S2A) was in much greater numbers. In addition, the morphology of these activated microglia with greatly enlarged somas and significantly reduced processes, and most were “tubular” or “worm-like” not spherical. Thus, they did not have the appearance of activated microglia normally associated with neurodegeneration.

The four animals sacrificed at the 1-day time point showed minimal or no clear classic evidence of Iba1 immunoreactive activated microglia in the septum or hippocampus (Table 1 and Additional file 1: Figure S1B) or the hippocampus or thalamus (Table 1 and Additional file 1: Figure S2B). This, despite the fact that in two of the animals, there was FJc evidence of significant neurodegeneration at the 1-day time point (Table 1), is a time point that significantly underestimates the actual number of neurons compared to the 3-day time point (Bowyer et al. unpublished, [26]). Only one of the four animals showed any early evidence microglia activation in the septum with either Iba1 or Cd11b [36] (biomarker for microglia) or soma enlargement (data not shown). In contrast, in the caudate putamen, all four of the animals showed clear evidence that microglial activation was underway at 1 day post METH (Additional file 1: Figure S2B) which has been previously reported by several laboratories [16, 37–39]. There was virtually no evidence in any brain region, in any of the four animals, examined at either the 1 or 3 days time points, of Iba1 or Cd11b immune-reactive macrophages invading the CNS or intercalating/invading into the brain vasculature.

**Fig. 3:**
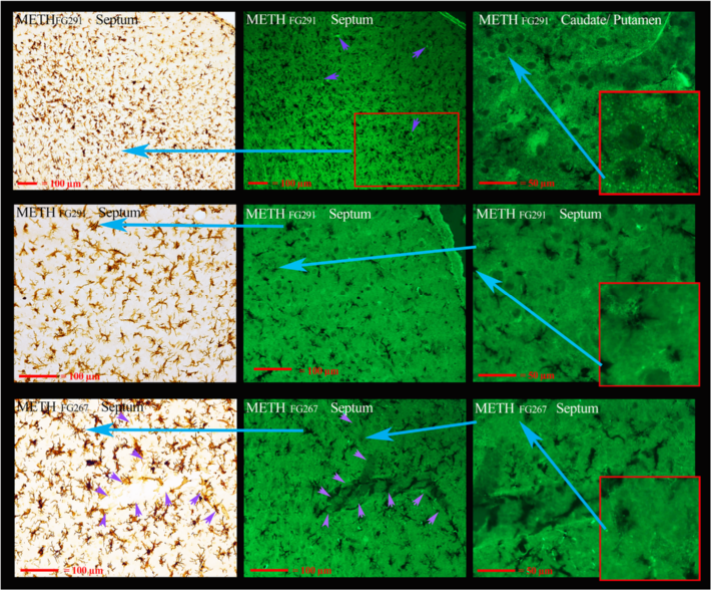
Pronounced microglia activation in the septum with little or no apparent neurodegeneration. Dual labeling of microglia with IBA1 immunoreactivity and FJc to detect neurodegeneration is shown in the dorsal and more lateral aspects of the septum. The three *left-hand panels* show the DAB-labeled microglia with incandescent illumination while the three *center*, and three most *right-hand panels* are fluorescent images obtained using a TRITC filter (for FJc detection of neurodegeneration). The *top left* and *center panels* from a METH-treated animal (3 days post dosing) show that there is pronounced microglia activation with minimal evidence of neurodegeneration. The most intensely labeled structures in these dorsal lateral regions, indicated by *purple arrows*, are vasculature-related. The *top right-hand panel* shows the FJc-labeled degenerating puncta in the caudate putamen at high magnification of the same animal. The remaining six panels are from two different METH-treated animals that further show there is little or no FJc evidence of neurodegeneration in many of the dorsal septal regions with activated microglia. The *purple arrows* in two of the bottom panels outline a large vessel that is present. The *blue arrows* show where the region of high magnification resides in the low magnification panels

FJc labeling in the cortex and particularly the thalamus revealed the loss of cell bodies (neurons) and their dendrites and axonal-like process/terminals (Table 1, Additional file 1: Figure S3A). In the ventral and more medial septum, there was evidence of FJc^+^ fibers of passage but very few (ventral medial septum) or no instances of FJc labeling indicating cell body loss (Additional file 1: Figure S3B). Dual labeling for Iba1 immunoreactivity and FJc indicated that most of the activated microglia immunoreactive for Iba1 that were present in the dorsal and central lateral septum were found in regions in which there was no FJc labeling (Fig. 3). In fact, there were areas in the central/mid-septum with significant FJc fiber labeling where the no juxtaposed activated microglia are found.

**Fig. 4:**
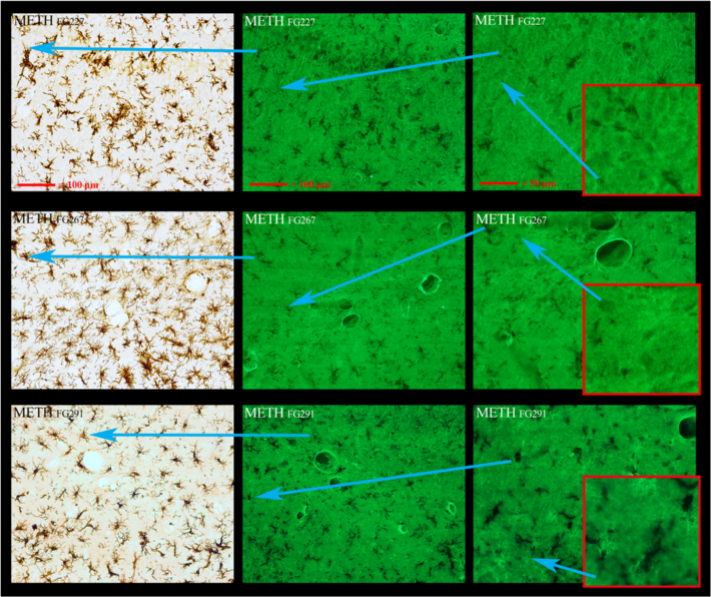
Pronounced microglia activation occurs with little or no apparent neurodegeneration. Dual labeling of microglia with IBA1 immunoreactivity and FJc to detect neurodegeneration is shown in the rostral and dorsal-medial area of the hippocampus. Pronounced microglial activation can be seen in the three *left-hand panels*, each for a different METH-treated animals (IDs present at the top left of panels). There was no evidence in this region in any of the three animals for terminal, axonal, or cell body labeling degeneration from FJc labeling in the remaining six panels. Again, as seen in the septum, the most intensely labeled structures were some of the vasculature present. The *blue arrows* show where the region of high magnification resides in the low magnification panels

There was little or no FJc labeling in the hippocampus that could be associated with any type of neurodegeneration in any of the animals (Additional file 1: Figure S4). Figure 4 shows dual labeling for FJc and Iba1 immunoreactivity in the anterior medial hippocampus as well. We modified the original methods of Schmued et al. [31] such that faint FJc labeling of other structures such as vasculature can be seen in some cases. Because of this, the fainter images of vasculature, probably endothelium, can be seen in Fig. 4. Note that in this figure, vascular-like structures faintly labeled with FJc appear juxtaposed to activated microglia.

**Fig. 5:**
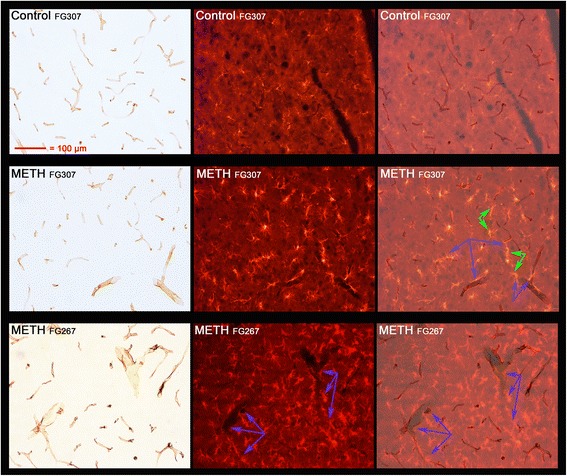
Activated microglia in septum associated with vasculature after METH. Septal sections from a control and two METH-treated animals were double labeled using DAB labeling of an antibody to RECA1 and TRITC labeling of an antibody to IBA1. The *far left panels* show the DAB-labeled RECA1 immunoreactivity to vasculature through visible light and the *middle panels* show the TRITC-labeled IBA1 immunoreactivity to microglia through fluorescent illumination. The three *far right panels* are a merger of the first two panels. The *purple arrows* show regions of particular interest (see the “Results” section for details). All panels are of the same magnification

**Fig. 6:**
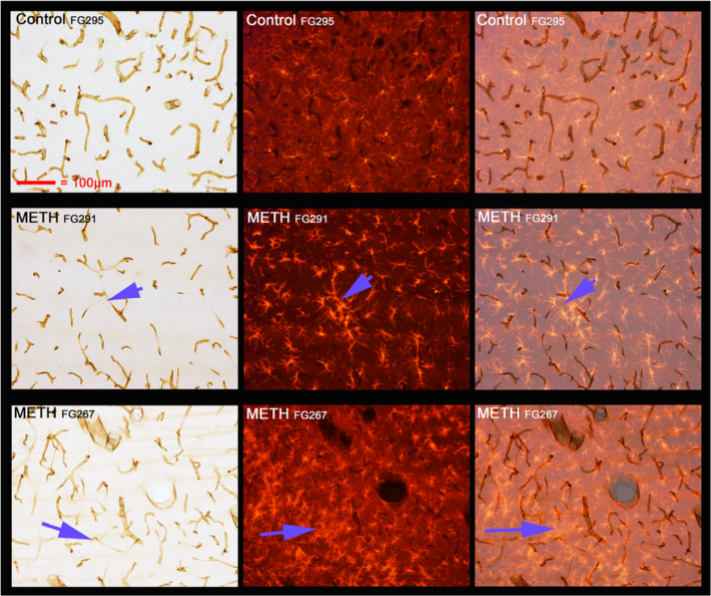
Activated microglia in hippocampus associated with vasculature after METH. Hippocampal sections from a control and two METH-treated animals were double labeled using DAB labeling of an antibody to RECA1 and TRITC labeling of an antibody to IBA1. The *far left panels* show the DAB-labeled RECA1 immunoreactivity to vasculature through visible light and the *middle panels* show the TRITC-labeled IBA1 immunoreactivity to microglia through fluorescent illumination. The *far right panels* are a merger of the first two panels. The *indigo arrows* show regions of particular interest (see the “Results” section for details). All panels are of the same magnification

**Fig. 7:**
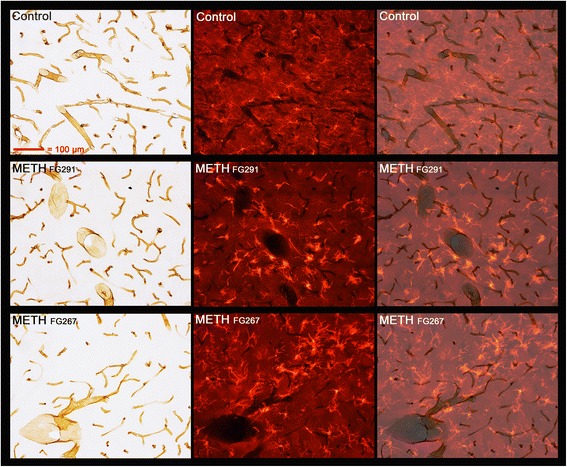
Activated microglia in the thalamus associated with vasculature after METH. Thalamic sections from a control and two METH-treated animals were double labeled using DAB labeling of an antibody to RECA1 and TRITC labeling of an antibody to IBA1. The *far left panels* show the DAB-labeled RECA1 immunoreactivity to vasculature through visible light and the *middle panels* show the TRITC-labeled IBA1 immunoreactivity to microglia through fluorescent illumination. The *far right panels* are a merger of the first two panels. The *indigo arrows* show regions of particular interest (see the “Results” section for details). All panels are of the same magnification

Double labeling to identify both vascular endothelia and microglia was performed to discern whether or not the activated microglia were associated with vasculature in the septum, hippocampus, and thalamus. This was accomplished using antibodies to two markers Cd11b and Iba1 for microglia and RECA-1 antibody for endothelia. Combinations of either TRITC-labeled Iba1 immunoreactivity and DAB-labeled RECA-1 immunoreactivity or DAB-labeled Cd11b immunoreactivity and nitro blue tetrazolium-labeled RECA-1 immunoreactivity to identify vascular endothelium show examples of the many close associations of endothelium with activated microglia in (Fig. 5, septum; Fig. 6, hippocampus; Fig. 7 VL/VM thalamus) 3 days after METH. In Fig. 6, the purple arrows show a region of very intense activation of microglia surrounding regions where vascular damage is intense in the hippocampus.

**Fig. 8:**
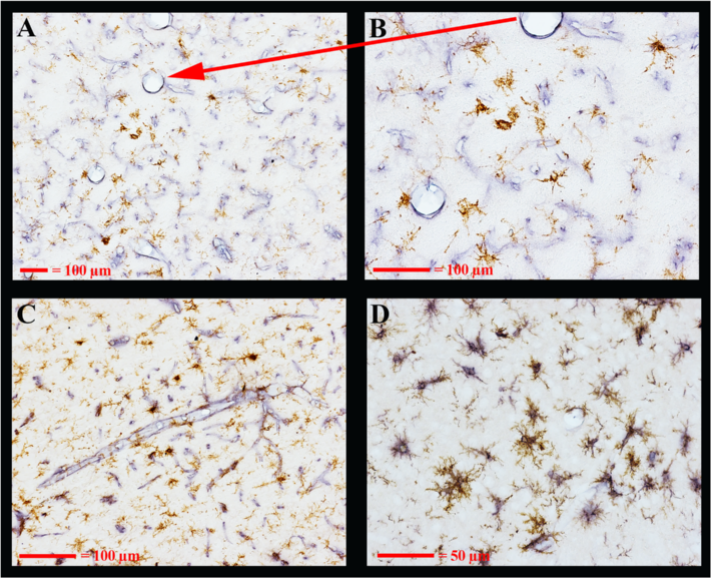
Activated microglia immunoreactive for Cd11b are shown in association with vasculature after METH. DAB immunolabeling of Cd11 to detect microglia and alkaline phosphatase/tetrazolium-immunolabeling of RECA1 were performed in sections of a METH animal. Shown are the associations of activated microglia with vasculature in the septum in panels **a** and **b** or the thalamus in panel **c** in visible light. Double labeling of microglia with DAB immunolabeling for Cd11b and alkaline phosphatase/tetrazolium-immunolabeling for IBA1 is shown in panel **d**. *Magnification bars* in lower left-hand corner

Double labeling with DAB-labeled Cd11b immunoreactivity for microglia and blue tetrazolium-labeled RECA1 for vasculature again shows the close association of many/most of the activated microglia with vasculature in both the septum (panels A and B) and the ventrolateral thalamus (panel c, Fig. 8). In panel D, the microglia can be seen double labeled with DAB-labeled Cd11b immunoreactivity and blue tetrazolium-labeled Iba1 immunoreactivity. There is a very close overlap in labeling between the two microglial markers. However, in the activated microglia, the Cd11b tends to be located in the regions of the microglia more distal to the cell body (more filamentous processes) while the IBA1 is more concentrated in the central regions of the microglia.

**Fig. 9:**
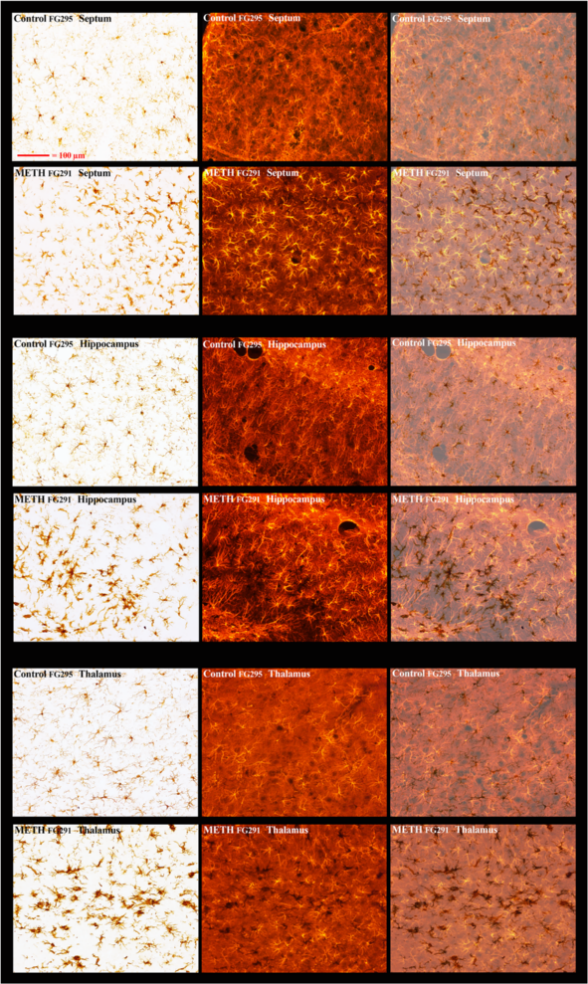
Astrocyte morphology in regions of METH-induced microglial activation. Dual labeling with DAB in conjunction with IBA1 immunoreactivity and TRITC for GFAP immunolabeling shows the morphology of astrocytes in the septum (top 6 panels), hippocampus (middle 6 panels), and thalamus (bottom 6 panels) in regions in conjunction with activated microglia after METH. The *left-hand column* of panels shows the IBA1-labeled microglia under incandescent illumination while the *center column* of panels shows the fluorescent images of the TRITC-labeled astrocytes. The *right-hand column* of panels is a merger of the incandescent and fluorescent images. All panels are of the same magnification (see the *upper left-hand panel* for magnification reference)

Figure 9a–c shows the morphology of astrocytes surrounding the activated microglia in the dorsal lateral septum, medial anterior hippocampus, and the VL/VM thalamus at 3 days after METH. The swollen and elongated activated DAB-IBA1-labeled microglia are clearly seen under incandescent light (remember from Fig. 8 that the DAB-IBA1 is not labeling the many of distal processes of these activated microglia). The astrocytes in these three brain regions appeared to be somewhat “swollen” and of a slightly different morphology. However, there are no discrete areas within any of the three regions where the morphology of the astrocytes juxtapose to the activated microglia is altered. More extensive data from DAB-GFAP labeling of astrocytes in the septum, hippocampus, and thalamus also indicate that there is a general mild hypertrophy in these regions, but there are no discrete areas within the regions where there is a distinctive change in astrocyte morphology (Additional file 1: Figure S9).

## Discussion

Much has been learned within the last 10 years about microglia including their origin and early integration into the newly developing brain [18, 40], role in both neurological disease [3, 19], and response to neurotoxic insults [2]. However, most of the research relating to microglia focuses on their interaction with neurons including the formation of synapses [19], their role in the repair and support of damaged neurons [3, 17], and their proposed responses resulting in adverse neuroinflammation [41]. However, it is clear from very recent research that much is still unknown about the role and importance of microglia in the adult brain under normal conditions or after neurotoxic insult [14, 22, 42–44]. The results of this investigation indicate that activated microglia associate closely with vasculature in regions such as the septum and hippocampus where there is minimal or no evidence of neurodegeneration after METH with the dosing paradigm we employed in the present experiments.

Microglia serve as microsensors of underlying pathology and are activated at sites of neural damage [45]. Previous research looking at microglial responses to METH and amphetamine toxicity have focused on regions of neurodegeneration, both axonal/terminal degeneration or neuronal (cell body) death [9–11, 13, 25, 39, 46]. In such research, we have noted that microglia have appeared to surround vasculature in regions of neurodegeneration in the hippocampus after METH [25]. However, in those studies, the METH exposure was severe enough to produce status epilepticus and significant neurodegeneration of pyramidal neurons (particularly in the CA2 and CA3 regions) as well as evidence of protracted BBB breakdown in the hippocampus. This left open the possibility that either microglia were merely responding to intense neurodegeneration or that macrophages had entered the brain in the regions of such severe damage and altered their morphology to resemble microglia. We have also seen similar effects in the thalamus with neurotoxic exposures to amphetamine where microglia appear to surround vasculature in the thalamus (unpublished data, [26]). Although there was little evidence of BBB disruption at this time point after amphetamine in this region, the microglia were observed in regions of significant neurodegeneration.

There is a significant literature related to amphetamine and METH with respect to adverse effects on brain vasculature and how this may be related to hyperthermia and neurotoxicity. Initially, it was observed that even a single dose of METH can produce small discrete regions of vascular leakage in several brain regions of laboratory animals when body temperatures exceed ≈41.6 °C [28, 47]. Some of these regions coincided with where amphetamines have been shown to produce neurotoxicity. A later study, using the same dosing paradigm as used in our present study (but substituting amphetamine for METH), observed the same discrete vascular leakage (≤1.0 mm in diameter) to occur throughout the entire drug exposure (multiple injections over time) [27]. This leakage lasted only briefly (less than 2 h) during the amphetamine administration. However, a single administration/injection of a very high dose of METH or amphetamine (40 mg/kg) can produce even longer lasting (6 to 12 h) and more extensive BBB leakage (in the septum, anterior medial hippocampus, and amygdala nuclei) that results in significant neurodegeneration in these regions [25, 26].

Much less is known with respect to how the vascular damage that is produced by amphetamines relates to the hypertension and reduced blood flow that may occur during exposure. A single dose of METH has been shown to produce prolonged reduced perfusion [48], which could certainly adversely affect neuronal function and lead to neurotoxicity. As well, neurotoxic exposure to amphetamine produces changes lasting for at least 1 day in the cortex, meninges, and associated vasculature indicating adverse vascular events have occurred in the cortical surface vasculature [49, 50]. Others have reported neurotoxicity and microglia activation in the caudate putamen after amphetamines that may be related to dopamine receptor interactions [51, 52], effects subsequent to vascular alterations, or damage [53]. Thus, in light of the preceding discussion, it is not entirely unexpected that microglia might respond to just vascular damage as well as neuronal damage.

Other studies have noted that microglia can be activated in the absence of underlying neurodegeneration, but these effects are still classified as neuroimmune responses (e.g., [14, 24]). Similar to those studies, evidence of neurodegeneration in the septum and hippocampus were not observed in the present study yet activated microglia surrounding vasculature in regions of the septum and hippocampus were seen after METH. Such activation was not seen in other regions of the forebrain except in two animals that had such activated microglia in the piriform cortex and amygdala (data not shown). The morphology of the activated microglia in septum and hippocampus was not that of enlarged spheres with radial processes that can be seen surrounding degenerating neurons but of a swollen, elongated tubular form with seemingly shrunken processes as stained with IBA1. However, double labeling immune-labeling with Cd11b indicated that the processes of these activated microglia may not have shrunk in size but that IBA1 levels in the processes were greatly reduced or not present and that Cd11b was present distally. As well, there is no evidence of BBB breakdown as determined by IgG immunereactivity or macrophage invasion in the brain either 1 day or 3 days post METH. Even in the ventrolateral thalamic nuclei and ventromedial thalamic nucleus, where significant neurodegeneration occurred, there were at least an equal number of the activated tubular-shaped microglia closely associated with vasculature.

Pericytes also play an important role in capillary flow, angiogenesis, blood-brain barrier, and immune responses, and they are very similar to microglia in transcriptome expression profile [51, 52]. Although some of the IBA1 expressing cells surrounding the vasculature seen in our studies may have been or be pericytes, the vast number observed in the hippocampus, septum, and thalamus would seem to be far too great to be just pericytes. Moreover, there morphology of most of these IBA1-positive cells does not seem to fit that reported for pericytes [53]. However, since there are no clear/certain histological markers that distinguish pericytes from microglia, we cannot definitively determine the percentage of pericytes that might also be IBA1 positive and associated with vasculature in these regions.

It is not clear how the astrocytes are interacting with the microglia in the septum, hippocampus, or thalamus that are activated 3 days after METH exposure. There appears to be a perceptible modest hypertrophy of the astrocytes in these three regions, but it is region-wide and not in conjunction with the specific areas of microglial activation surrounding the vasculature. Areas with modest toxicant-induced neural damage can result in increases in levels of the astrocytic protein, GFAP, in the absence of an apparent astrocytic hypertrophy [38], an effect often preceded by microglia activation in the same region [38]. Thus, it is possible that a small degree of METH-induced damage, undetectable via FJc, in these areas, is responsible for the observed modest astrocyte responses. However, they do not explain the close responses/associations of microglia with specific vasculature and not neurons.

## Conclusions

The simplest and, in our opinion, the most logical explanation for the presence of activated microglia surrounding the vasculature in two discrete regions of the brain where there is no neurodegeneration would be that the microglia are responding to METH-induced vascular damage. This was facilitated by multiple episodes of hyperthermia (an exposure comparable to heat stroke) and hypertension that was produced by the METH exposure. It thus follows that when more episodes of hyperthermia occur, the vascular damage and microglial activation in the septum and hippocampus are more likely to occur and more intense. The METH exposure we employed only produces transient vascular disruptions lasting less than 1 to 2 h and is most commonly seen in the thalamus, septum, piriform cortex, and medial ventral hippocampus [26–28]. This may be sufficient enough to allow cytokines normally excluded from the brain or the damage-associated proteins (DAMPs) produced by METH exposure [54] to enter the brain and activate the microglia. On the other hand, it may be that adversely affected brain vascular endothelia are directly signaling the microglia through some unknown messenger(s) signaling pathway. If this is the case, then the term cerebrovascular-induced brain inflammation would be a more appropriate characterization rather than describing it as neuroinflammation. It follows that detection of such activated microglia several days after an “adverse” event occurring in brain could be used as a biomarker indicating that some vascular damage or “irritation” had occurred.

## Additional files

Additional file 1:
**Figure S1A.** DAB-Iba1 labeled microglia activation in the septum at 3 days after METH exposure. **Figure S1B.** DAB-Iba1 labeled microglia in the septum and hippocampus at 1day after METH exposure. **Figure S2A.** DAB-Iba1 labeled microglia in the hippocampus at 3 days after METH exposure. **Figure S2B.** DAB-Iba1 labeled microglia in the cudate/putamen at 1 day after METH exposure. **Figure S3A.** Coincidence of degenerating (FJc+) neurons and activated microglia in the thalamus at 3 days after METH. **Figure S3B.** FJc-labeling in the septum at 3 days after METH exposure. **Figure S4.** FJc-labeling in the medial hippocampus at 3 days after METH exposure. **Figure S9.** DAB-GFAP labeled astrocytes in the medial hippocampus at 3 days after METH exposure. (PDF 15680 kb)

## Abbreviations

AMPH: amphetamine
a.k.a. Cd11b: integrin, alpha M (complement component 3 receptor 3 subunit), integrin, alpha M (complement component 3 receptor 3 subunit) a.k.a.Itgam
DAB: diaminobenzedine
FITC: fluorescein isothiocyanate
FJc: Fluoro-Jade C
allograft inflammatory factor 1: Aif1 a.k.a Iba1
METH: methamphetamine
RECA-1: rat endothelial cell antigen-1
TRITC: tetramethylrhodamine-isothiocyanate
