# Supplementary material for: Vascular-directed responses of microglia produced by methamphetamine exposure: indirect evidence that microglia are involved in vascular repair?
Source: J Neuroinflammation. 2016 Mar 12;13:64. doi: 10.1186/s12974-016-0526-6 (PMC4789274; doi:10.1186/s12974-016-0526-6)
Supplement: Additional file 1: — Figure S1A. DAB-Iba1 labeled microglia activation in the septum at 3 days after METH exposure. Figure S1B. DAB-Iba1 labeled microglia in the septum and hippocampus at 1day after METH exposure. Figure S2A. DAB-Iba1 labeled microglia in the hippocampus at 3 days after METH exposure. Figure S2B. DAB-Iba1 labeled microglia in the cudate/putamen at 1 day after METH exposure. Figure S3A. Coincidence of degenerating (FJc+) neurons and activated microglia in the thalamus at 3 days after METH. Figure S3B. FJc-labeling in the septum at 3 days after METH exposure. Figure S4. FJc-labeling in the medial hippocampus at 3 days after METH exposure. Figure S9. DAB-GFAP labeled astrocytes in the medial hippocampus at 3 days after METH exposure. (PDF 15680 kb) [file 12974_2016_526_MOESM1_ESM.pdf]

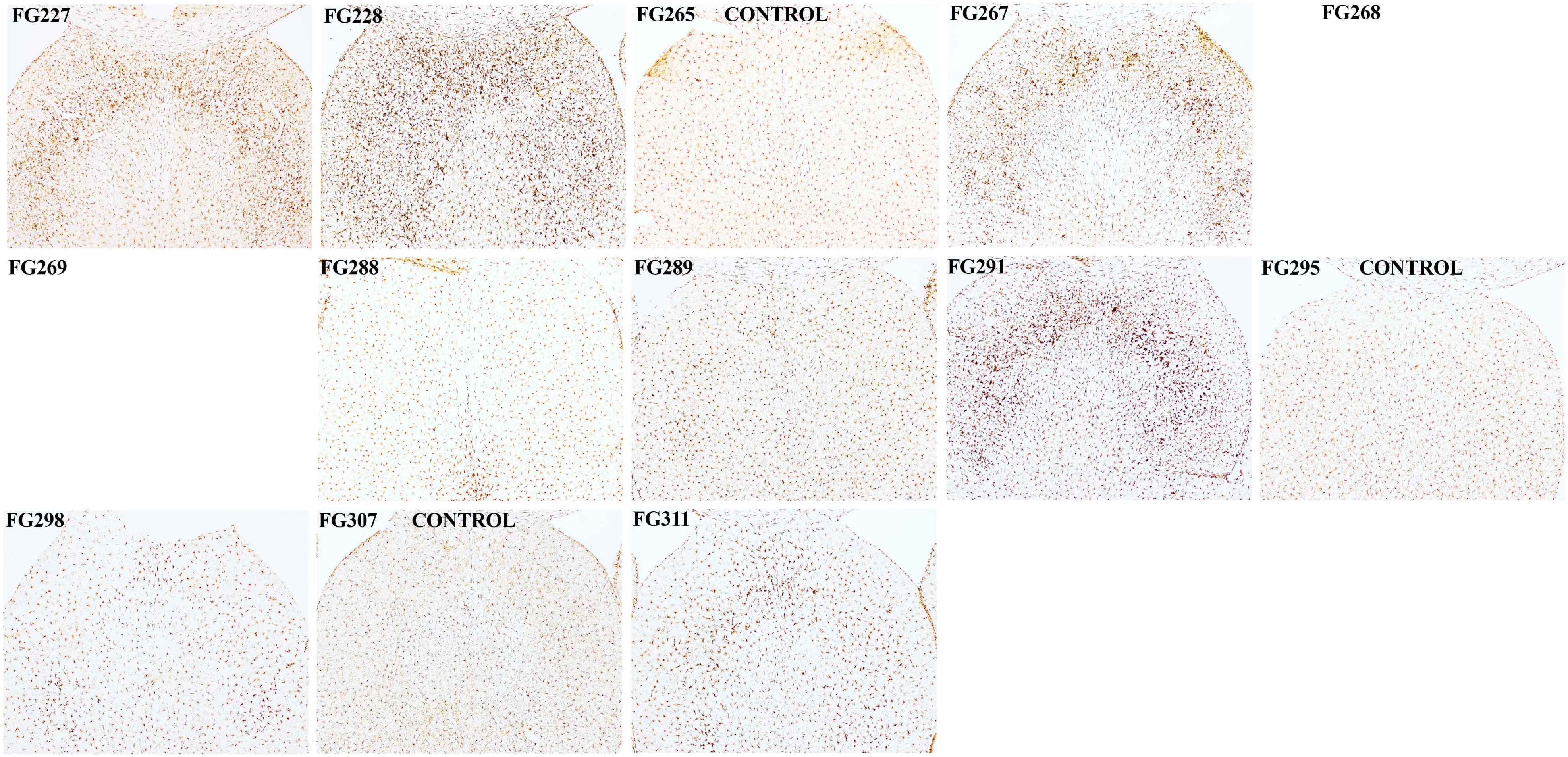
FG227
FG228
FG265 CONTROL
FG267
FG268
FG269
FG288
FG289
FG291
FG295 CONTROL
FG298
FG307 CONTROL
FG311

Supplemental Figure 1B

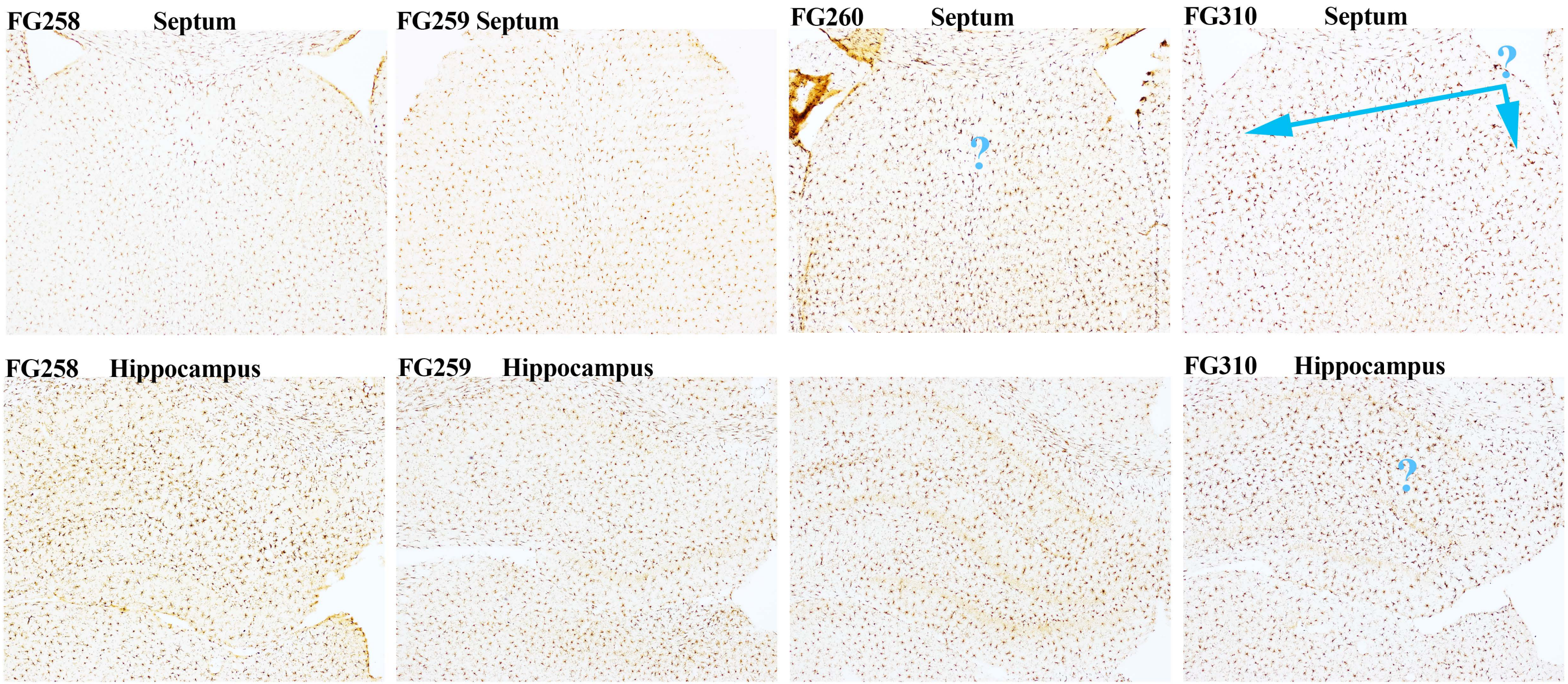

Question marks in figure indicate that the overall size of the soma of the microglia may have increased. However, the shape/ morphology of the microglia are similar to control. The arrows in the septum of FG310 point to regions where the shape/ morphology of the microglia are more like those associated with vasculature in regions where vascular disruption likely occurred.

Supplemental Figure 2A

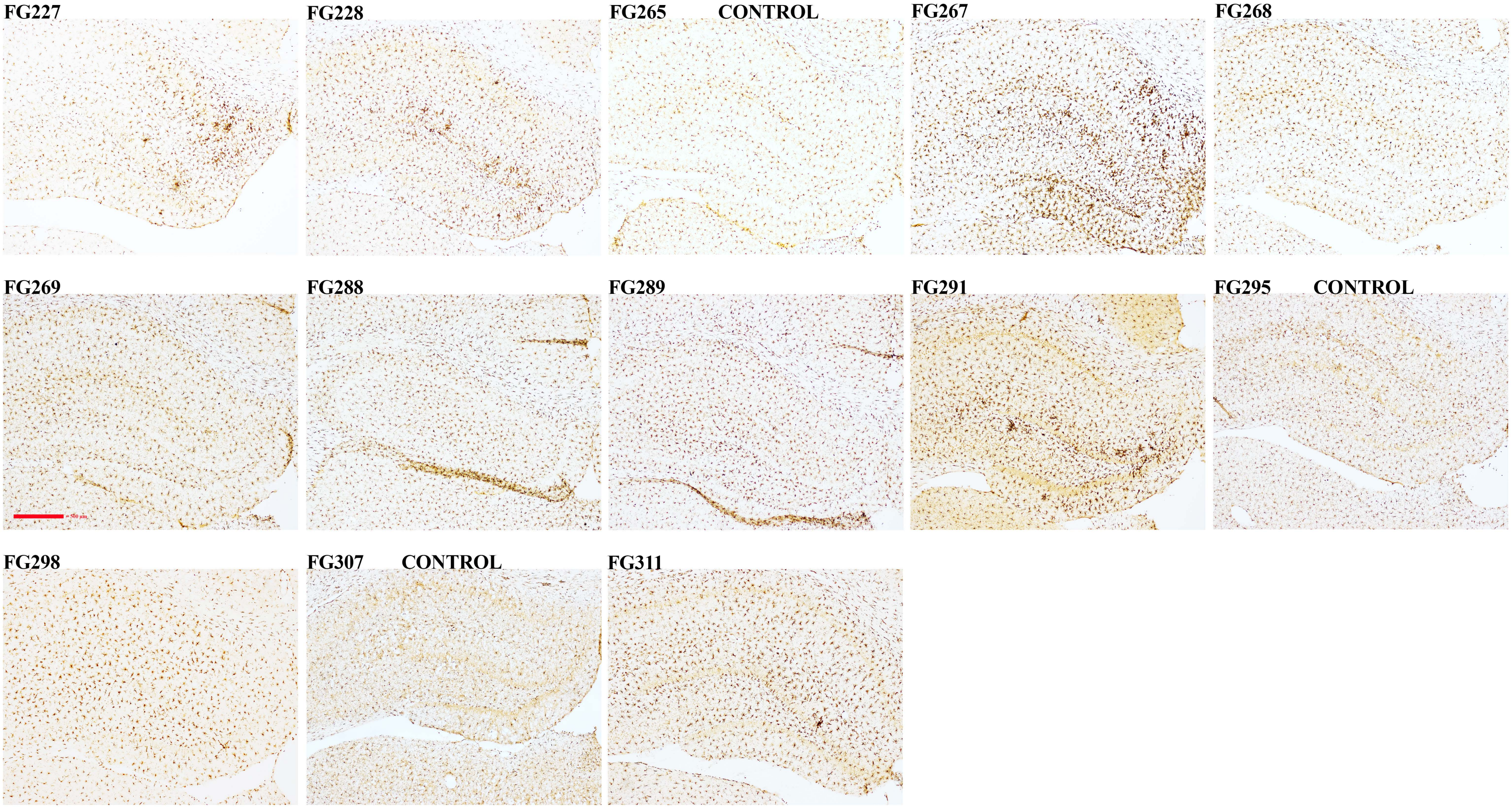

Supplemental Figure 2B

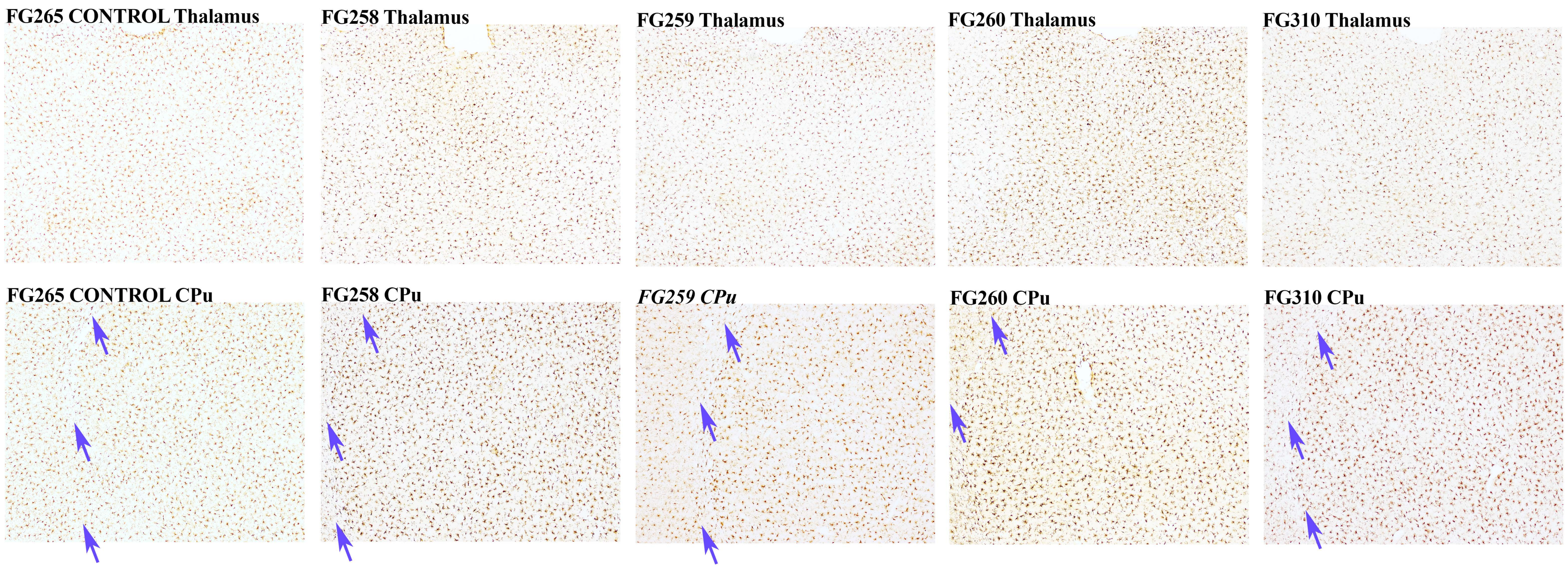

Arrows indicate the position of the corpus colosum situated between the cortex and the lateral CPu. Note that in the METH treated rats that the microglia are larger in the CPu than the cortex, which is not the case in the control.

Supplemental Figure 3A. Bowyer et al.

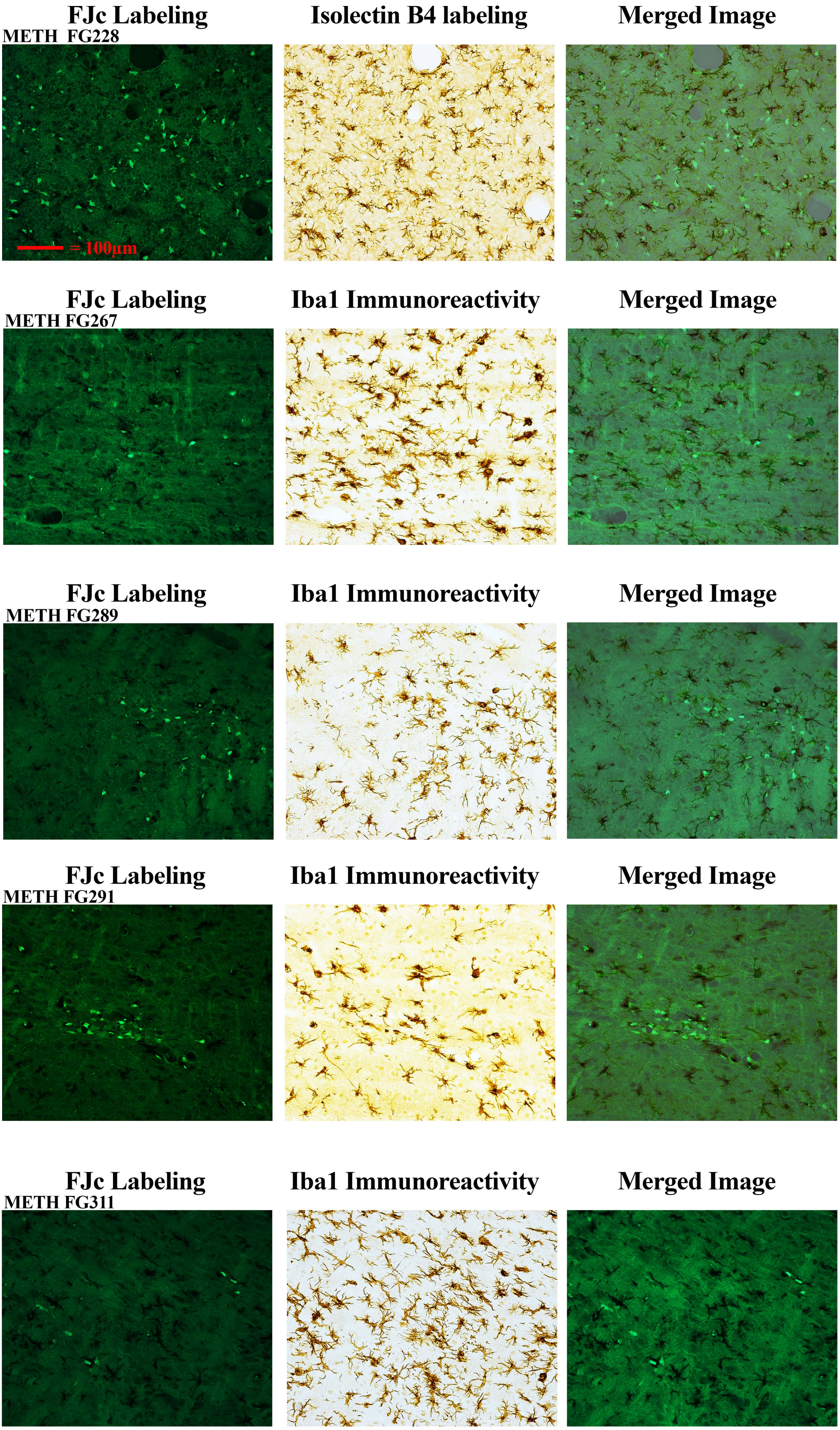

Neurodegeneration in the ventrolateral and or ventromedial thalamic nuclei was detected via FJc-labeling in the left-most column of panels while microglia activation is seen with DAB-Iba1 immunolabeling in the middle column of panels at 3 days after METH. The merger of the two images is shown in the right-most column of panels. Magnificaion is the same for all the panels shown. See magnification bar in top left panel.

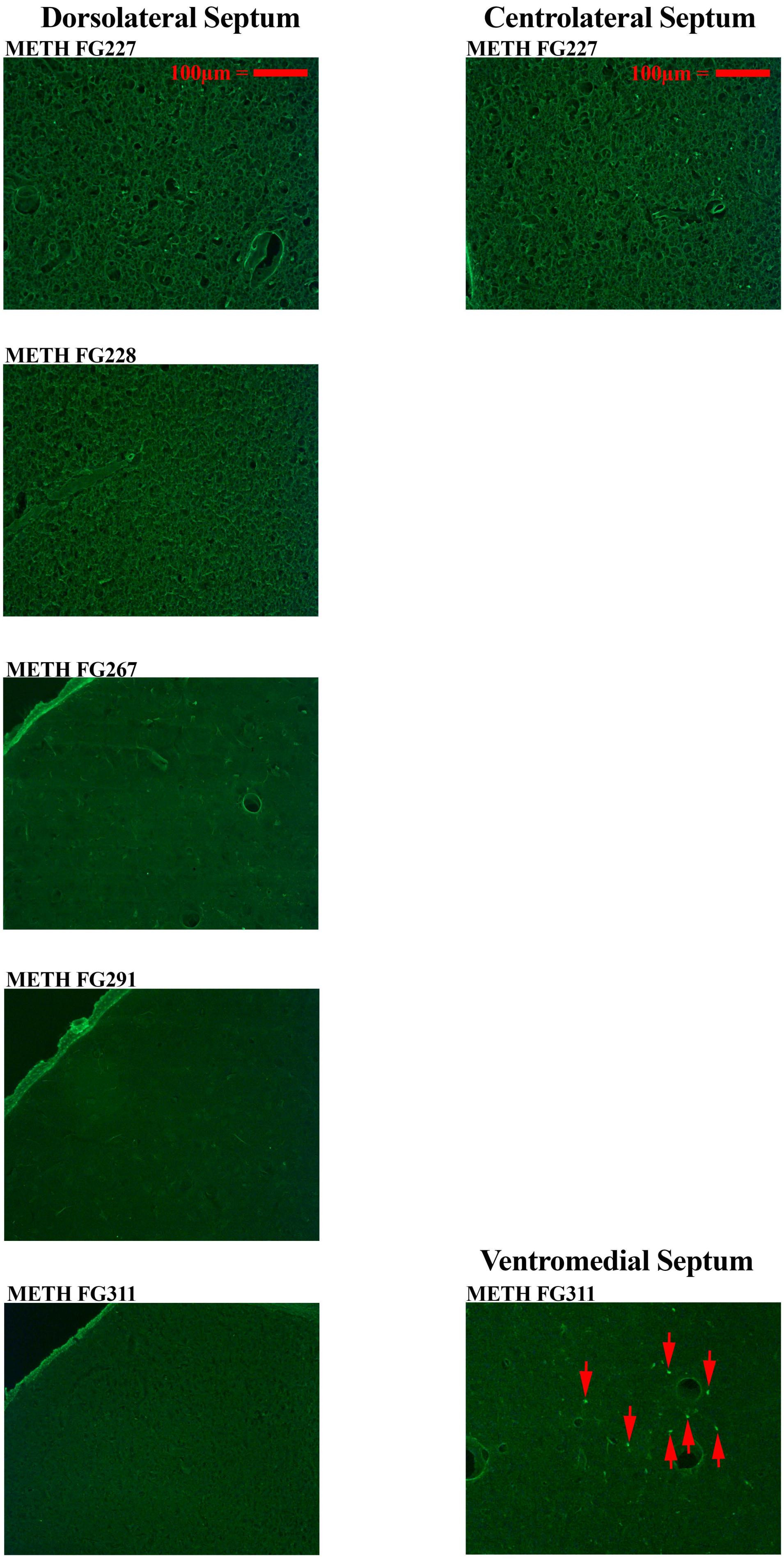

FJc images of the dorsolateral septum are shown in the left-most column at 3 d after METH. There is faint labeling in the FG227 rat that is most likely due to vasculature and not degenerated axons or dendrites the FJc labeling in the ventromedial septum of FG311 may be due to degenerating axons or dendrites. All panels are of the same magnifications (see upper-most panels).

Supplementary Figure 4. *Bowyer et al.*

METH FG227

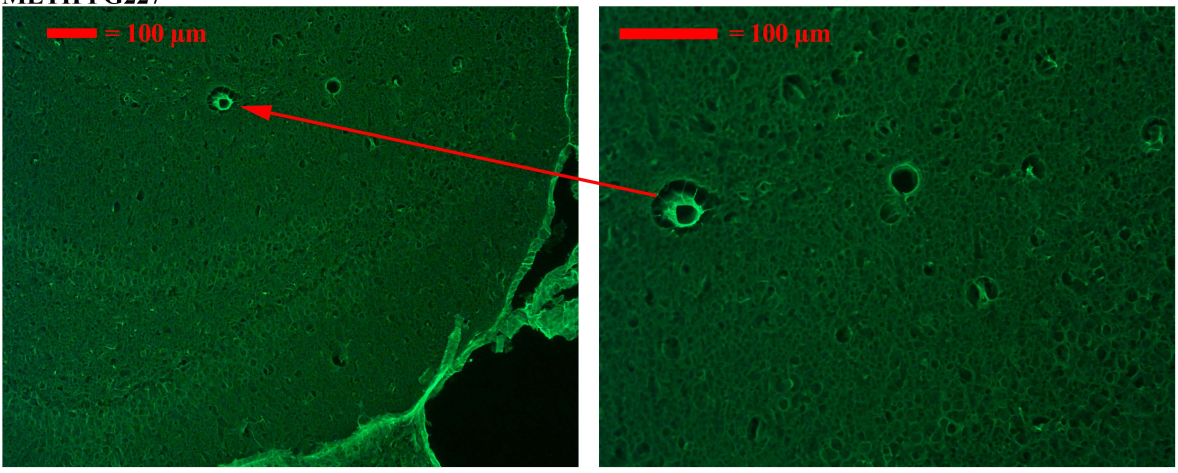

METH FG228

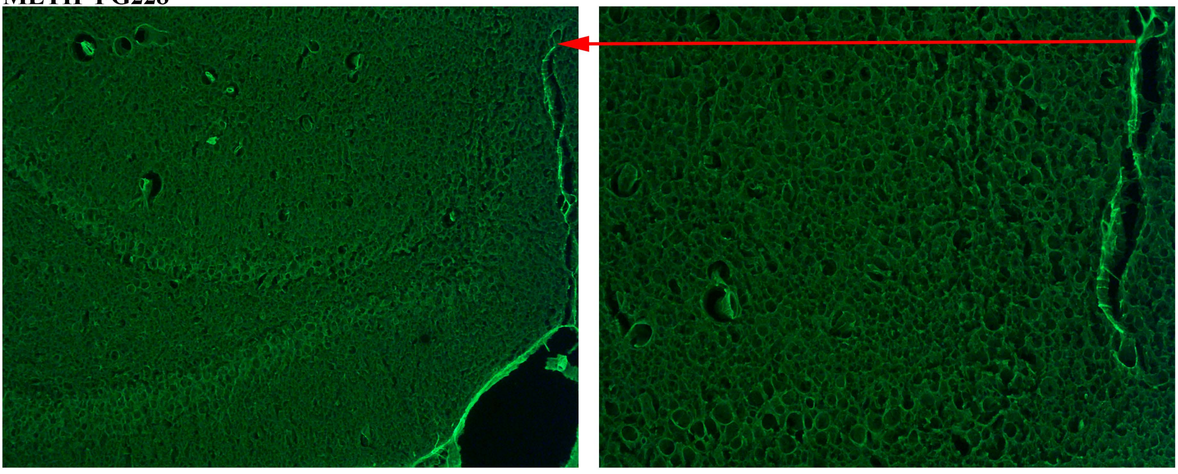

METH FG267

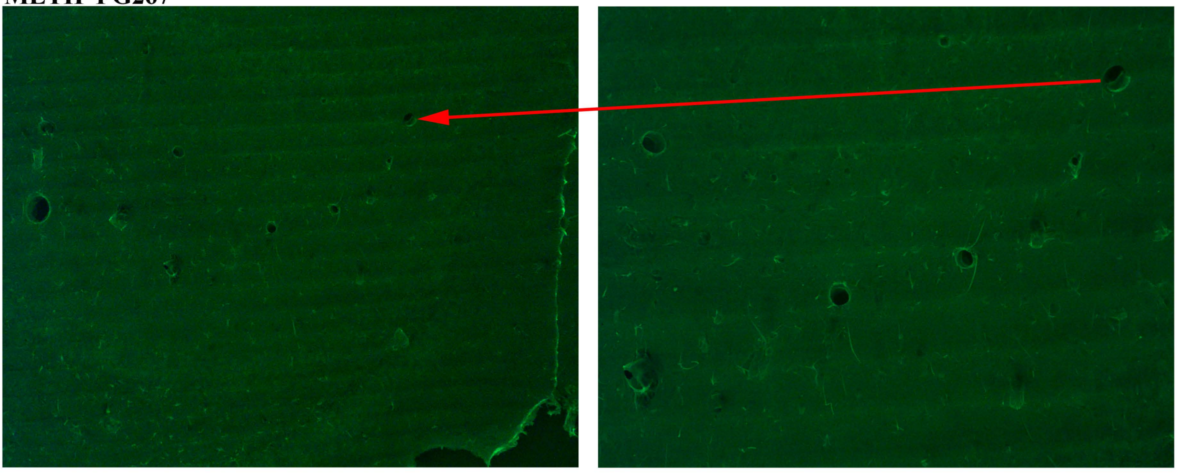

METH FG291

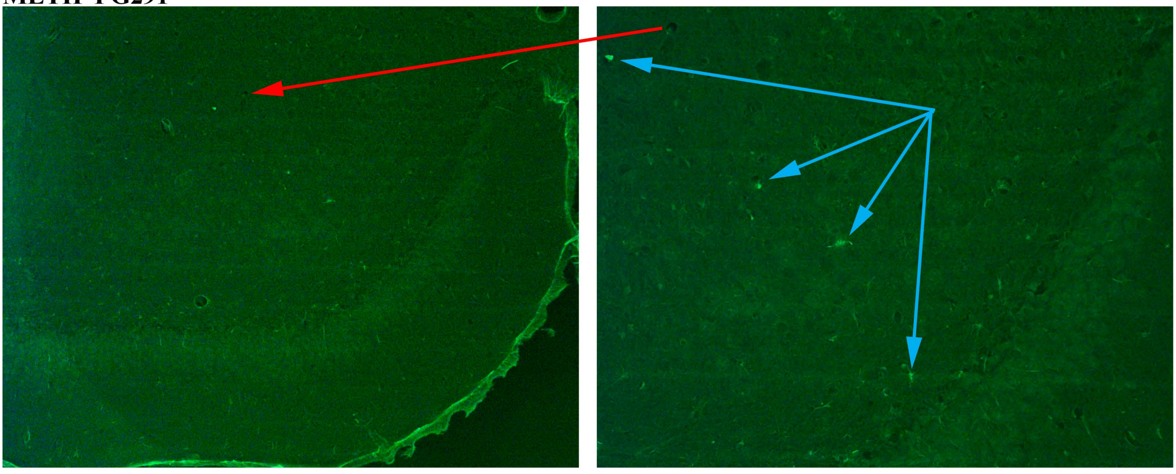

METH FG311

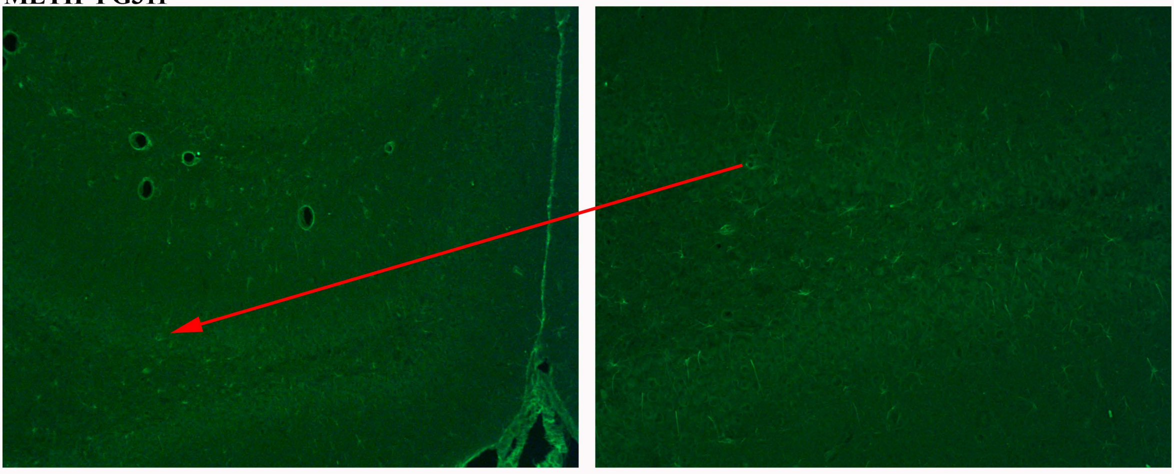

FJc labeling in the anterior medial hippocampus at two different magnifications at 3 d after METH. The red arrows are for orientation of two magnifications of the same area. The blue arrows in FG291 appear to be related to vasculature. Note that in FG311 ghost like structures that are not neuroal in origin are seen. These may or may not be faint labeling of astrocytes. It is important to note that none of the FJc labeled structures would be visable/ labeled if the more stringent methods of of Schmued et al would have been used.

Supplemental Figure 9. Bowyer et al.

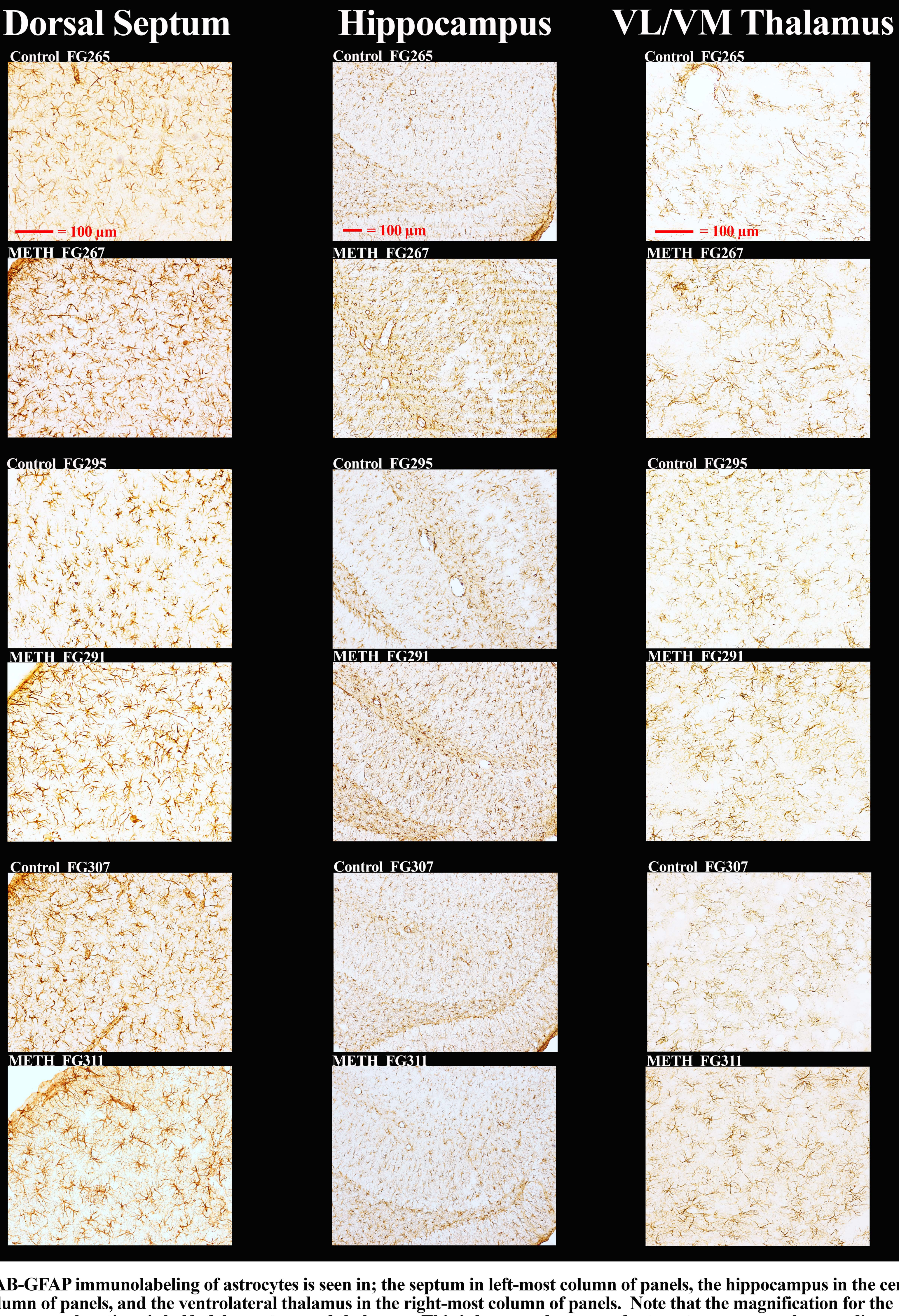

DAB-GFAP immunolabeling of astrocytes is seen in; the septum in left-most column of panels, the hippocampus in the cente column of panels, and the ventrolateral thalamus in the right-most column of panels. Note that the magnification for the hippocampal sections is half of the septum and thalamus. This is because the types of astrocytes are so much more diverse in the hippocampus and not well represented at the higher magnification. Magnifications for each panel in the column are the
